# Correction to ‘The relationship between psychosocial job stressors and insomnia: The mediating role of psychological capital’

**DOI:** 10.1002/nop2.70785

**Published:** 2026-10-22

Du, Juan , Shi‐Zhe He , Min‐Jie Li , et al. 2023. “The Relationship between Psychosocial Job Stressors and Insomnia: The Mediating Role of Psychological Capital.” Nursing Open. 10.1002/nop2.1693.36929116

Following publication of this article, the authors identified errors in the reporting of the study's data‐collection period and the number of questionnaires distributed.

The data analyzed in this study were collected during the first wave of data collection, conducted between June and August 2019. Owing to an error during manuscript preparation, the manuscript reported the timing and questionnaire distribution figures from a subsequent wave of data collection rather than the initial wave from which the analyzed dataset was derived.

The correct number of questionnaires distributed was 720 rather than 810. The number of valid questionnaires included in the analyses remains 658, and therefore the analyses, results, and conclusions of the article are unchanged.

In addition, the authors requested correction of the author‐contribution information. Juan Du and Shi‐Zhe He contributed equally to this work and should be recognized as co–first authors.

The authors apologize for these errors and confirm that the corrections do not affect the findings or conclusions of the article.

